# Discrimination due to sexual orientation and oral health-related quality of life among adolescents

**DOI:** 10.1590/1807-3107bor-2024.vol38.0085

**Published:** 2024-09-13

**Authors:** Bruno EMMANUELLI, Jessica Klöckner KNORST, Orlando Luiz do AMARAL-JÚNIOR, Maria Laura Braccini FAGUNDES, Jessye Melgarejo do Amaral GIORDANI, Thiago Machado ARDENGHI

**Affiliations:** (a) Universidade Federal de Santa Maria – UFSM, School of Dentistry, Department of Stomatology, Santa Maria, RS, Brazil.

**Keywords:** Adolescent, Oral Health, Quality of Life, Sexual Behavior

## Abstract

To evaluate the association between discrimination based on sexual orientation and oral health-related quality of life (OHRQoL) in adolescents. This was a cross-sectional study nested in a cohort performed in southern Brazil. The baseline assessment was carried out in 2010 with a sample of preschoolers (1 to 5 years). Subsequently, these individuals were reassessed, and for the present study, only the data from the final follow-up in 2020 were considered. OHRQoL was assessed by the short version of the Child Perceptions Questionnaire 11-14 (CPQ11-14). The discrimination due to sexual orientation was measured using item 10 of the Olweus Bully/Victim Questionnaire. Sociodemographic (sex, age, skin color, maternal education, household income) psychosocial (sense of coherence), and clinical variables (untreated dental caries) were also evaluated. Multilevel Poisson regression analysis was performed to verify the associations. Results are present as rate ratio (RR) and 95% confidence interval (95% CI). A total of 429 adolescents were evaluated – about 67.1% of those assessed at baseline. The prevalence of discrimination due to sexual orientation was 3.3%. Adolescents who reported suffering episodes of discrimination due to sexual orientation presented overall CPQ11-14 scores 16% higher (RR 1.16, 95%CI 1.01-1.36) than their counterparts. Adolescents who reported suffering episodes of discrimination due to sexual orientation presented poorer OHRQoL.

## Introduction

Oral health currently emerges as a positive concept that encompasses the essential attributes for maintaining the well-being and quality of life of individuals.^1^ In this sense, oral health-related quality of life (OHRQoL) has been considered an important measure of oral health. The concept of OHRQoL is multidimensional and generally reflects how individuals perceive their oral health in terms of carrying out their daily activities, interaction and social well-being.^2^

The literature has consistently suggested the role that social determinants such as income, education and social capital, as well as oral clinical determinants, as untreated dental caries and gingivitis, can play in the deterioration of OHRQoL.^3-6^ Currently, the focus has shifted towards intrinsic factors that were previously overlooked by societies for a long time. These factors include various forms of oppression and discrimination, which have been found to be closely related to oral health outcomes. It is important to acknowledge and address these factors in order to improve overall oral health and promote healthcare equality.^7-9^.

In this sense, one type of discrimination that has been brought out of the shadow into the light is the discrimination based on sexual orientation.^10,11^ This kind of discrimination is also known as gender-based discrimination, which involves treating someone unfavourably because of their gender, encompassing their sexual orientation. Sexual orientation is defined as an inherent or immutable enduring emotional, romantic, or sexual attraction to other people, and is independent of gender identity.^12^ In general, sexual orientation has been defined as orientation towards someone of the same sex (lesbian/gay), the opposite sex (heterosexual), both (bisexual), all types of sexual genders (male, female, transgender, or intersex), or the lack of sexual attraction to another person (asexuality).^10,12^

Previous studies have evidenced that individuals that face this type of discrimination are more likely to present poor mental health and worst quality of life.^13,14^ Other studies also demonstrated that discrimination due to sexual orientation has been related to substance abuse among adolescents and adults.^11,15^ Hence, it has been underscored that the discrimination stemming from sexual orientation might be intricately connected with health behaviour, and the state of emotional and social well-being. In this context, it is hypothesized that episodes of this type of discrimination can also affect aspects related to oral health, especially during adolescence, a period of innumerable changes, identity encounters and adaptations to social structures.^16^ However, to the best of our knowledge, no previous study has evaluated this relationship. Thus, considering the relevance of the topic in this life stage, this study aimed to evaluate the relationship between the occurrence of discrimination due to sexual orientation and OHRQoL among adolescents. We hypothesized that adolescents who suffered this type of discrimination are more likely to present poorer OHRQoL.

## Methods

### Ethical aspects

This study was previously approved by the Research Ethics Committee of the Federal University of Santa Maria (protocol CAAE 11765419.1.0000 5346). All individuals and their caregivers agreed to participate and signed an informed consent.

### Study design and sample

This was a cross-sectional study nested in a cohort carried out in the city of Santa Maria, southern Brazil. The baseline sample assessment was performed in 2010 on children’s Multivaccination Day. All children who attended the vaccination event aged 1 to 5 years were considered eligible and systematically selected in primary health units around the city. A total of 15 primary health units were selected (from a total of 28), distributed throughout the eight administrative regions of the city. In each of them, for every fifth child in the vaccination line, one was invited to take part in the study. Subsequently, the individuals were reassessed in 2012, 2017, and 2020. Although the data are part of a cohort study, this study only considered data from the last follow-up (2020), justified by the inclusion of new variables in the survey that allowed a comprehensive analysis of discrimination and sexual orientation. The data collection period ranged from November 2019 to January 2021, with an interruption due to the COVID-19 pandemic (between March and October 2020).

Data were collected by a trained team using tools and indices recommended by previous literature. The team consisted of dentists and undergraduate students, totalling twelve evaluators. Adolescents were assessed in their respective schools or at home, according to data recorded at baseline. Some adolescents and their respective families were also contacted primarily by telephone or social media (Facebook, Instagram and WhatsApp). More details about the epidemiological survey have been previously published.^6,17^

Currently, Santa Maria has an estimated population of 271,633 inhabitants, of which approximately 19,833 are aged between 10 and 15 years.^18^ To satisfy the sample requirements, we performed sample power calculation. The calculation considered a mean difference in overall Child Perceptions Questionnaire 11-14 (CPQ_11-14_) scores of 15.1 (SD 8.6) in exposed adolescents (who suffered discrimination due to sexual orientation) and of 11.0 (SD 8.4) in unexposed adolescents (who did not suffer discrimination due to sexual orientation) and a 95% confidence interval, resulting in sample power of 93%.

### Variables

To assess the OHRQoL, the outcome of the study, the adolescents answered the short version of the Child Perceptions Questionnaire 11-14 (CPQ_11-14_), validated for Brazilian adolescents.^19,20^ CPQ_11-14_ has 16 questions, subdivided into 4 domains: oral symptoms, functional limitation, social well-being, and emotional well-being. Answers are provided on a Likert-type scale from 0 to 5 points: “never” = 0; “once or twice” = 1; “sometimes” = 2; “often” = 3; and “every day/almost every day” = 4. The final score is obtained by the sum of all items, and ranges from 0 to 64 points. The higher the score obtained, the poorer the OHRQoL. For data analysis purposes, the variable was used quantitatively through overall scores.

The discrimination due to sexual orientation was assessed using item 10 of the Brazilian version of the Olweus Bully/Victim Questionnaire (OBVQ).^21^ The question was “In the last month, I have been humiliated because of my sexual orientation or grimace?”, with the scored response options reflecting the frequency of behaviours: “never” (score 0); “once or twice in the last month” (score 1); “once or several times a week” (score 2)”. For data analysis, it was considered the absence (score 0) or presence (scores 1 or 2) of perceived discrimination due to sexual orientation.

Some variables were used as possible confounders of the association. Demographic characteristics included sex (boys or girls), age (categorized by the mean – 12 years), and skin color, collected using IBGE criteria (black, brown, white, indigenous and yellow) and later dichotomized into white and non-white.^23^ These categories were used due to the limited representation of certain categories of non-whites, being commonly used in literature. Socioeconomic variables were maternal education, recorded in years of formal education and categorized as < 8 years or ≥8 years, and household income, reported in Brazilian reals (US$1.00 is equivalent to approximately R$ 5.0) and later categorized into quartiles of income, from quartile 1 – ‘Lowest income’ to quartile 4 – ‘Highest income’.

For adjustment, we also considered the adolescents’ sense of coherence (SOC), assessed by the Brazilian version of the SOC-13 scale,^23^ which is responded on a 1- to 5-point Likert-type scale, with scores ranging from 13 to 65. Subsequently, the SOC was dichotomized by the mean (36.2) into low (< 36) or high (≥ 36). Dental caries was recorded by the International Caries Detection and Assessment System (ICDAS).^24^ The ICDAS scores range from 0 to 6 and evaluate sound surfaces (score 0), white spot lesions (scores 1 and 2), enamel lesions (scores 3), shadow lesions (score 4), and dentine lesions covering less than half (score 5) or more than half the surface (score 6).^24^ For data analysis, ICDAS was dichotomized into absence (scores 0, 1, 2 and 4) or presence (scores 3, 5 and 6) of cavitated lesions. Cavitated lesions were considered as untreated dental caries. The intra- and inter-examiner agreement for dental caries assessment was greater than 0.7 (kappa statistics).

### Data analysis

The data were analysed using the STATA 14 program (StataCorp LLC). A descriptive analysis of the sample was performed. Comparison between followed and dropout individuals to verify the representativeness of the sample overtime was performed using the chi-square test and the t-test. The outcome considered was overall CPQ_11-14_ scores. The analysis of sample characteristics according to overall CPQ_11-14_ was also performed.

Unadjusted and adjusted multilevel Poisson regression analysis was performed to verify the association between discrimination due to sexual orientation and overall CPQ_11-14_ scores. Demographic, socioeconomic, psychosocial, and oral clinical variables were used for model adjustments according to theoretical aspects related to OHRQoL.^2^ The multilevel structure used a fixed effects model with a random intercept, where adolescents (first level) were nested in their respective neighbourhoods (second level). Results are presented as rate ratio (RR) and 95% confidence interval (95%CI).

## Results

A total of 429 adolescents were assessed (about 67.1% from baseline). There were no significant differences in the variables considered in the study between followed and dropout individuals from the original cohort sample (p > 0.05).

Table 1 presents the descriptive characteristics of the adolescents. The sample was balanced between boys and girls, age, and skin color. About 50.2% were girls, 56.5% had 12 years or more, and 51.5% were non-whites. Most adolescents were from families in the two lowest income quartiles, and 30.4% had mothers with less than 8 years of formal education. About half of the sample had a low SOC (51.7%). The prevalence of discrimination due to sexual orientation was 3.3%. The overall CPQ_11-14_ score of sample was 11.1 (SE 0.6).

**Table 1:** Main characteristics of the sample, Santa Maria (n= 429).

Variables	n(%)*
Socidemographic chacracteristics	
Sex	
Boys	209 (49.8)
Girls	220 (50.2)
Age (years)	
< 12	189 (43.5)
≥ 12	240 (56.5)
Skin color	
White	215 (48.5)
No-white	211 (51.5)
Maternal education	
≥ 8 years of formal education	285 (69.6)
< 8 years of formal education	110 (30.4)
Household income in R$	
Quartile 1 Lowest	110 (29.2)
Quartile 2	79 (23.4)
Quartile 3	108 (25.4)
Quartile 4 Highest	77 (21.9)
Psychosocial characteristics	
Sense of choerence	
Low	222 (51.7)
High	207 (48.3)
Discrimination due to sexual orientation	
No	413 (96.7)
Yes	14 (3.3)
Oral health measure	
Untreated dental caries	
Absent	300 (69.4)
Present	128 (30.6)
Outcome	Mean (SE)*
OHRQoL	11.1 (0.6)

*Taking into account the sampling weight; R$, Brazilian Real (US$1.00 is equivalent to R$5.0 approximately); SE, standard error; OHRQoL, oral health-related quality of life.

Sample characteristics according to overall CPQ_11-14_ scores are displayed in Table 2. Regarding sexual orientation discrimination, overall CPQ_11-14_ scores were 15.1 (SE 3.2) versus 10.1 (SE 0.6) among adolescents who reported and did not report discrimination, respectively. Overall CPQ_11-14_ scores were also higher in girls (11.9 [SE 0.9]), adolescents with low SOC (14.1 [SE 1.0]), and those who presented untreated dental caries (13.1 [SE 13.0]).

**Table 2:** Sample characteristics according to overall CPQ11-14 scores (n = 429).

Variables	CPQ11-14
	Mean (SE)*
Socioemographic chacracteristics	
Sex	
Boys	10.4 (0.8)
Girls	11.9 (0.9)
Age (years)	
< 12	11.7 (0.7)
≥ 12	10.7 (0.9)
Skin color	
White	11.8 (1.0)
No-white	10.5 (0.6)
Maternal education	
≥ 8 years of formal education	11.2 (0.8)
< 8 years of formal education	11.2 (0.9)
Household income in R$	
Quartile 1 Lowest	12.2 (0.9)
Quartile 2	12.6 (1.7)
Quartile 3	9.6 (0.7)
Quartile 4 Highest	11.3 (1.6)
Psychosocial characteristics	
Sense of choerence	
Low	14.1 (1.0)
High	8.3 (0.6)
Discrimination due to sexual orientation	
No	11.0 (0.6)
Yes	15.1 (3.2)
Oral health measure	
Untreated dental caries	
Absent	10.3 (0.6)
Present	13.0 (1.3)

*Taking into account the sampling weight; R$, Brazilian Real (US$1.00 is equivalent to R$5.0 approximately); SE, standard error; CPQ, child perception questionnaire.

Table 3 shows unadjusted and adjusted association between discrimination and overall CPQ_11-14_ scores. In the unadjusted analysis, all variables considered were significantly associated with the outcome (p < 0.05). In the adjusted analysis, adolescents who reported discrimination due to sexual orientation presented overall CPQ_11-14_ scores 16% higher than their counterparts (RR 1.16; 95% CI 1.08–1.24). Girls present higher scores in CPQ_11-14_ (RR 1.07 95%CI 1.01–1.14). Older age had protective effect on OHRQoL (RR 0.92; 95%CI 0.86–0.98). Low maternal education and low income were associated with higher CPQ_11-14_ scores. Considering the psychosocial variable, individuals with high SOC presented 46% lower overall CPQ_11-14_ scores than their counterparts with low SOC (RR 0.54; 95%CI 0.51–0.58). The presence of untreated dental caries was associated with 16% higher CPQ_11-14_ scores (RR 1.16 95% CI 1.08–1.24).

**Table 3:** Unadjusted and adjusted association between occurrence of discrimination due to sexual orientation and overall CPQ11-14 scores

Variables	Unadjusted	Adjusted	p–value
	RR (95% CI)	RR (95% CI)	
Sociodemographic chacracteristics			
Sex			< 0.01
Boys	1 (reference)	1 (reference)	
Girls	1.23 (1.16–1.30)	1.07 (1.01–1.14)*	
Age (years)			0.015
< 12	1 (reference)	1 (reference)	
≥ 12	0.93 (0.88–0.99)	0.92 (0.86–0.98)*	
Skin color			0.037
White	1 (reference)	1 (reference)	
No–white	1.07 (1.01–1.14)	1.04 (0.97–1.15)	
Maternal education			0.021
≥ 8 years of formal education	1 (reference)	1 (reference)	
< 8 years of formal education	1.08 (1.01–1.15)	1.08 (1.01–1.15)*	
Household income in R$			< 0.01
Quartile 1 Lowest	1 (reference)	1 (reference)	
Quartile 2	0.88 (0.81–0.96)	0.90 (0.83–0.99)*	
Quartile 3	0.69 (0.64–0.75)	0.75 (0.68–0.82)*	
Quartile 4 Highest	0.79 (0.72–0.86)	0.89 (0.80–0.98)*	
Psychosocial characteristics			
Sense of choerence			< 0.01
Low	1 (reference)	1 (reference)	
High	0.55 (0.50–0.56)	0.54 (0.51–0.58)*	
Discrimination due to sexual orientation			< 0.01
No	1 (reference)	1 (reference)	
Yes	1.28 (1.11–1.48)	1.16 (1.01–1.36)*	
Oral health measure			
Untreated dental caries			< 0.01
Absent	1 (reference)	1 (reference)	
Present	1.16 (1.09–1.24)	1.16 (1.08–1.24)*	

RR: rate ratio; CI: confidence interval *p < 0.05; R$, Brazilian Real (US$1.00 is equivalent to R$5.0 approximately).

## Discussion

This study evaluated the association between discrimination due to sexual orientation and OHRQoL in adolescents. Our findings showed that adolescents who suffered this type of discrimination presented poorer OHRQoL, confirming our conceptual hypothesis. Although some studies have evaluated the impact of discrimination due to sexual orientation on other health outcomes,^13,14^ its impact on oral health outcomes had not yet been explored.

The fact that adolescents who faced sexual orientation discrimination had a poorer OHRQoL can be explained through different theories.^25^ First, individuals who experience discrimination tend to have greater emotional and mental effects, such as anxiety disorders and high levels of depression.^13,26^ In this context, high levels of psycho-emotional stress can negatively impact health habits^25^ and lead to negative self-perception,^14^ which can interfere with the perception of one’s oral health and quality of life. In addition, stress responses may be related to participation in unhealthy groups,^25^ which translates into higher consumption of alcohol, tobacco and drugs, affecting oral health and quality of life at this stage of life.^27,28^

The prevalence of discrimination due to sexual orientation was 3.3% in our sample. A prior study conducted in the USA demonstrated a prevalence of sexual discrimination of about 32% at the age of 20 years, with a decreasing trend with advancing age.^29^ Another study carried out with homosexuals in twelve states in Brazil, demonstrated a prevalence of 65% self-reported discrimination due to sexual orientation.^30^We attribute the disparities in prevalence compared to our data primarily to factors such as sample size, data collection methods, the inclusion of individuals exclusively representing non-heterosexual sexual orientations in other studies, and variations in the age groups under investigation.

Other aspects could also explain the difference in the prevalence of sexual discrimination. We hypothesize that cultural aspects of the countries where the studies were conducted may affect this result. While discrimination remains prominent throughout Brazil,^30^ the topic has been widely discussed in recent years within various contexts, including the school environment.^9,31^ Variations in discrimination findings could also be due to the age of participants, which reflects the different moments of their lives. Entering the labor market between late adolescence and early adulthood may be even more challenging for LGBTQIA+ individuals, which would explain a higher prevalence of discrimination at this phase.

Our results also demonstrated that girls, younger age, low maternal education and income, low SOC level, and presence of untreated dental caries were also associated with poorer OHRQoL. These findings are consistent with previous studies conducted in adolescents.^5,32,33^ Among these findings, it is worth mentioning the positive relationship between SOC and OHRQoL. Previous studies have demonstrated that SOC plays an important role in adolescence. Being a factor that refers to resilience and self-management, high SOC acts as a protective factor against stress and has a positive impact on subjective oral health outcomes.^9,34^ In this way, SOC can be an important ally in improving oral health in this population, especially victims of discrimination. In addition, our results also highlighted the role of low socioeconomic status in OHRQoL, which is evidenced by several previous studies.^4,5,32^ In this context, low-income individuals are more susceptible to several risk factors that affect their health, such as less access to services, fewer material resources, and greater psychosocial stress,^4,5,32^ which directly impacts oral health and perceived quality of life.

This study has some limitations. First, data on sexual orientation discrimination were self-reported and assessed using a straightforward question, potentially constraining the precision of its assessment. However, this question was taken from a validated questionnaire with international coverage.^35^ Furthermore, given that the questionnaires were administered through interviews, some adolescents may have felt embarrassed to fully respond certain questions, which could have implications for the accurate reporting of the prevalence of sexual orientation discrimination. In this sense, there were no clarifications regarding the conceptual difference between sex and gender, which may have caused a response bias in the study. Finally, this was a cross-sectional study nested within a cohort, which may limit the representativeness of the sample since approximately 30% of the adolescents were dropouts over time. However, a sensitivity analysis demonstrated that there were no significant differences between the followed and dropout individuals throughout the cohort waves.

Despite the limitations, some strengths of the study should be highlighted, such as the assessment of an important predictor of oral health of individuals during their adolescence. Previous studies in the Brazilian context reported the occurrence of different types of discrimination among dental students around the country,^36^ and these episodes have also been present within the school environment.^9,32^ Studying these factors in this population is extremely important, as it affects general and oral health, the consequences of which can be perpetuated throughout the life cycle.

All these findings suggest that a multidisciplinary action should be taken by different health and education professionals to combat discrimination and promote oral health and well-being among LGBTQIA+, since the prejudice that they suffer can have an impact on their well-being and quality of life. Therefore, public health managers must be aware of these findings to promote strategies in this regard, in addition to those already covered at a national level. Future studies are suggested to explore this relationship in adolescents from other regions and also longitudinal studies should be conducted to test more deeply the causality and possible pathways among these factors.

## Conclusion

Our findings demonstrated that adolescents who reported suffering from discrimination due to their sexual orientation presented greater negative impacts on OHRQoL. This knowledge is important so that preventive measures to avoid this type of discrimination can be developed, aiming to improve the well-being and quality of life of this population.
